# Alkyl-Chitosan-Based Adhesive: Water Resistance Improvement

**DOI:** 10.3390/molecules24101987

**Published:** 2019-05-23

**Authors:** Narimane Mati-Baouche, Cédric Delattre, Hélène de Baynast, Michel Grédiac, Jean-Denis Mathias, Alina Violeta Ursu, Jacques Desbrières, Philippe Michaud

**Affiliations:** 1 Clermont Auvergne University, CNRS, SIGMA Clermont, Institut Pascal, F-63000 Clermont-Ferrand, France; narimane.mati@univ-rouen.fr (N.M.-B.); cedric.delattre@uca.fr (C.D.); helene.de_baynast@uca.fr (H.d.B.); michel.grediac@uca.fr (M.G.); alina-violeta.ursu@sigma-clermont.fr (A.V.U.); philippe.michaud@uca.fr (P.M.); 2 Normandie Univ, UNIROUEN, Glyco-MEV EA 4358, Glycobiology and Plant Molecular Matrix, EA 4358, 76000 Rouen, France; 3 Normandie Univ, UNIROUEN, SFR NORVEGE, 76000 Rouen, France; 4 IRSTEA, Laboratory of Engineering for Complex Systems, 9 avenue Blaise Pascal, CS 20085, 63178 Aubière, France; jean-denis.mathias@irstea.fr; 5 Université de Pau et des Pays de l’Adour (UPPA), IPREM, UMR 5254 CNRS/UPPA, Helioparc Pau Pyrénées, 2 avenue P. Angot, 64053 PAU CEDEX 09, France

**Keywords:** chitosan, alkylation, adhesive, water resistance

## Abstract

A chemical modification by grafting alkyl chains using an octanal (C8) on chitosan was conducted with the aim to improve its water resistance for bonding applications. The chemical structure of the modified polymers was determined by NMR analyses revealing two alkylation degrees (10 and 15%). In this study, the flow properties of alkyl-chitosans were also evaluated. An increase in the viscosity was observed in alkyl-chitosan solutions compared with solutions of the same concentration based on native chitosan. Moreover, the evaluation of the adhesive strength (bond strength and shear stress) of both native and alkyl-chitosans was performed on two different double-lap adherends (aluminum and wood). Alkyl-chitosans (10 and 15%) maintain sufficient adhesive properties on wood and exhibit better water resistance compared to native chitosan.

## 1. Introduction

Commercially available synthetic adhesives are mostly derived from depleting petrochemical resources. They present many advantages, such as high bond strength and water resistance [1]. However, most of them are composed of volatile organic compounds or other toxic compounds, which are very dangerous for health and environment, such as epichlorohydrin and formaldehyde [2,3]. The need for environmentally friendly and healthy adhesives represents a real challenge and pushes wood industries and academics to develop alternatives to synthetic adhesives. Among bio-based molecules/polymers which can provide those characteristics, polysaccharides are interesting candidates. Their numerous hydroxyl groups can interact with numerous chemical functions and their high molecular weight can provide cohesive strength to materials.

Chitosan (CS), a polysaccharide of the aminoglucopyran family obtained by alkaline deacetylation of chitin, is the only bio-sourced cationic polysaccharide. Soluble in acidic aqueous solutions (acetic acid, formic acid, etc.), it is composed of a randomly distributed β-(1,4)-linked d-glucosamine and *N*-acetyl-d-glucosamine. Chitosan has primary amine groups at the C-2 position, primary alcoholic groups at the C-6 position, and also secondary hydroxyl groups. This biopolymer is insoluble at neutral and basic pH due to intra and/or intermolecular hydrogen bonding, which form crystalline domains. Its degree of crystallinity depends on the degree of deacetylation (DD) and varies from 60 to 100%. Its good bonding and mechanical properties, its biocompatibility, and its biodegradability open the way to numerous original applications in the adhesive and binder fields [2,3,4,5]. For example, its performance as adhesive for wood, plant particles, glass, and aluminium surfaces was highlighted in the literature [2,4,5,6,7]. However, the main drawback of using chitosan as adhesive is its limited performance in aqueous environment, which restricts its application and durability.

Thanks to chitosans reactive sites, many grafting and/or modifications such as alkylation, hydroxyalkylation, carboxyalkylation, sulfation, thiolation, phosphorylation, acylation, and acidic or enzymatic depolymerizations can be performed under mild reaction conditions to create new functionalized derivatives [4,5,8,9,10,11]. Chitosan may be chemically modified by reductive amination leading to grafting hydrophobic alkyl chains along the hydrophilic macromolecular chain. The reductive alkylation procedure can be employed with equal facility for attaching non-saccharidic residues such as aldehydes and ketones to chitosan. This method seems to offer the advantages of simplicity and versatility in tailoring solubility and hydrophobicity [8]. This allows obtaining amphiphilic polymers with hydrophobicity (or hydrophile–lipophile balance) adjustable according to the length of the grafted alkyl chain or the degree of substitution (DS) of the amine functions [9]. Chitosans (CS) that are substituted with alkyl chains having a minimum of six carbon atoms demonstrate hydrophobic interactions in solution [9,12]. Moreover, the application of chitosan alkyl derivatives as rheological modifiers was studied by Desbrieres in 2004 [9]. Several applications are possible for those derivatives especially in aqueous solutions-based formulations such as paints, oil recovery, cosmetics, and food [13,14,15]. For example, [15] synthesized alkylchitosan has been used as paper coating material to improve water barrier properties.

However, the adhesive properties correlated with mechanical strength and water resistance of high-molecular-weight alkylated CS on wood and aluminium adherends have not been reported until now to the best of our knowledge. In this paper, we propose to modify chitosan by grafting an alkyl chain using an octanal (C8) and to evaluate/characterize its potential use as a water-resistant “green” adhesive for wood and aluminium surfaces.

## 2. Results

The adhesive properties of high-molecular-weight chitosan alkylated by an octanal (C8) with different degrees of substitution (10 and 15%) were investigated in this study with the aim of evaluating its potential use as “green” water-resistant adhesive. Therefore, different approaches were used to achieve this objective, ranging from the synthesis of alkyl-chitosan, its structural characterization by ^1^H-NMR analyses, the formulation of the adhesives based on alkyl-chitosans, and the assessment of their flow and adhesive properties to, finally, the determination of their water resistance.

### 2.1. Confirmation of Grafting on Chitosan

The degree of grafting/alkylation or degree of substitution (DS) in modified chitosan was determined using ^1^H-NMR analyses in D_2_O/HCl (pH~4) at 353 K [12].

**Figure 1:**
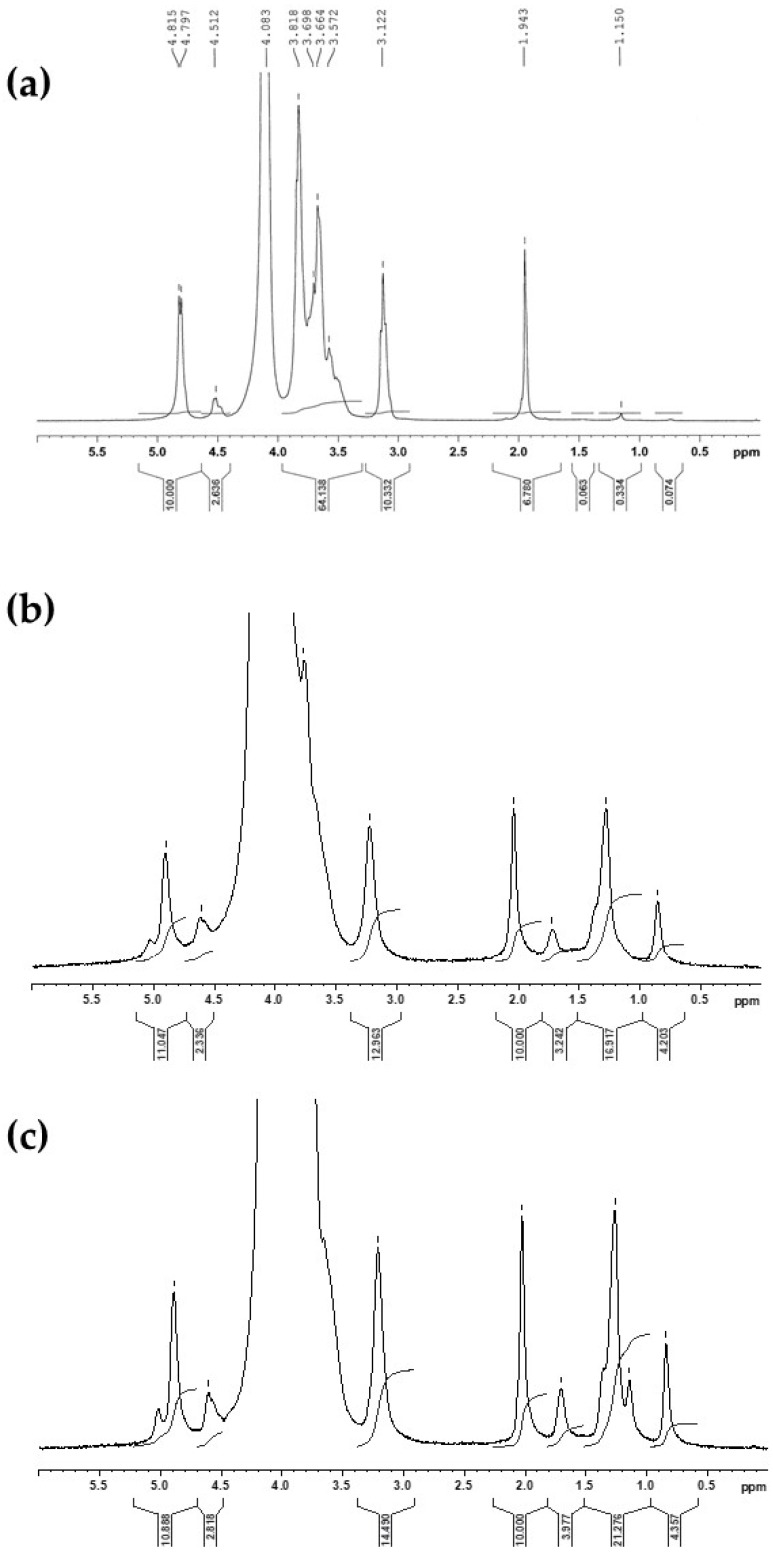
^1^H-NMR analyses of native chitosan (**a**), alkyl-chitosan with a degree of substitution (DS) of 10% (**b**) and DS of 15% (**c**) from an octanal (C8).

The ^1^H-NMR spectra (Figure 1) of *N*-alkyl-chitosan display characteristic peaks in the 1.7–0.9 ppm region, attributed to the protons of the methyl (-CH_3_) and methylene (-CH_2_-) groups grafted onto the chitosan chain, which also supports the chemical modification of chitosan. Moreover, it is possible to identify the different H-1 signals [12]:

* at 4.51 ppm, the acetylglucosamine unit,

* at 4.80 ppm, the unsubstituted glucosamine unit,

* at 4.94 ppm, the monosubstituted glucosamine unit.

The ^1^H-NMR spectra provide information that can be used to evaluate the acetylation degree (DA) of the original chitosan and the substitution degree (DS) of chitosan derivatives. The signals at 0.87 ppm and 1.63 ppm may be attributed to the -CH_3_ and -CH_2_ groups linked to the N atom, respectively. The degree of substitution was calculated as previously described [12], by comparing the integral of -CH_3_ signal at 0.87 ppm with the total of integrals from H-1 signals between 4.5 and 5 ppm. The DS were evaluated to be 10% (Figure 1b) and 15% (Figure 1c) for the different modified chitosans.

### 2.2. Evaluation of Bonding Properties of Alkyl-Chitosan

The bonding properties of chitosan (CS) and two alkyl-chitosans (CS-C8 10% and CS-C8 15%) formulated with acetic acid solutions (1 to 2% (*v/v*)) at different concentrations (2, 4, and 6% (*w/v*)) were evaluated after drying on two different adherends, wood and aluminium. The results are summarized in Table 1. The bond strength of chitosan and alkyl-chitosans were higher on the wood adherend compared with the aluminium one. Different mechanisms/theories of adhesion are suggested for chitosan/wood adherends and chitosan/aluminium adherends to explain this result. In fact, we assume that both systems have predominantly “mechanical adhesion”, occurring due to the penetration of the adhesive in irregular surface, asperities, pores, or micro-cavities. Wood has natural micro-cavities, and we created an irregular surface on the aluminium surfaces by chemical treatment to potentiate adhesion. Moreover, another complementary mechanism of adhesion is probably occurring in the chitosan/wood system, the “adsorption adhesion mechanism”, occurring when there are inter-molecular forces between adhesive and adherend such as those between the amine groups of chitosan and the polyphenolic compounds of the wood (lignin).

The results (Table 1) are in good agreement with those obtained by Patel et al. [2] in terms of formulated native and high-molecular-weight chitosan on pine wood adherend, supporting the good reproducibility of properties of chitosan. The loss of bonding properties was observed when the alkylation of chitosan occurred, which was expected with the addition of hydrophobic interactions. The best bond strength “σ” (MPa) performances for CS, CS-C8 10% and 15% (Table 1) were obtained with the concentration of 4% (*w/v*).

We can distinguish various modes of break or failure according to the place where the crack takes place during the mechanical analysis. For example, when the failure is on one of the substrates or on the adhesive, it corresponds to a cohesive failure, meaning that the adhesion between the constituents is the strongest. However, when the failure is at the interface, it is an adhesive failure.

For native chitosan, the mode of failure was structural, which means that the adhesive and the interface (wood/adhesive) were stronger than the wood adherend. For alkyl-chitosan, the mode of failure observed was mixed, namely cohesive and adhesive, which corresponds to a break in adhesive or in the wood/adhesive interface. For treated aluminium specimens, the mode of failure observed was cohesive.

### 2.3. Water Resistance Improvement

From the results exposed above, the water resistance of the best bonded systems was evaluated. We selected the formulated native and modified chitosans at the concentration of 4% (*w/v*) with 1% acetic acid. After immersion, a strong decrease in bond strength was observed for wood specimens after the wet condition tests (Table 2). Native chitosan had a bonding resistance divided by 10 (2.55 vs. 0.28 MPa). However, CS-C8 (DS of 15%) conserved its bonding properties (0.7 MPa), which was very encouraging in terms of the water resistance property. Moreover, referring to the literature, Patel et al. [2] obtained the best bond strength at 0.55 MPa on wood after test immersion in their best chitosan formulation, and Yamada et al. [4] obtained from a modified adhesive based on chitosan, the best bond strength at 0.4 MPa on glass surfaces.

### 2.4. Rheological Properties of Best Formulated Chitosan Adhesive

**Figure 2:**
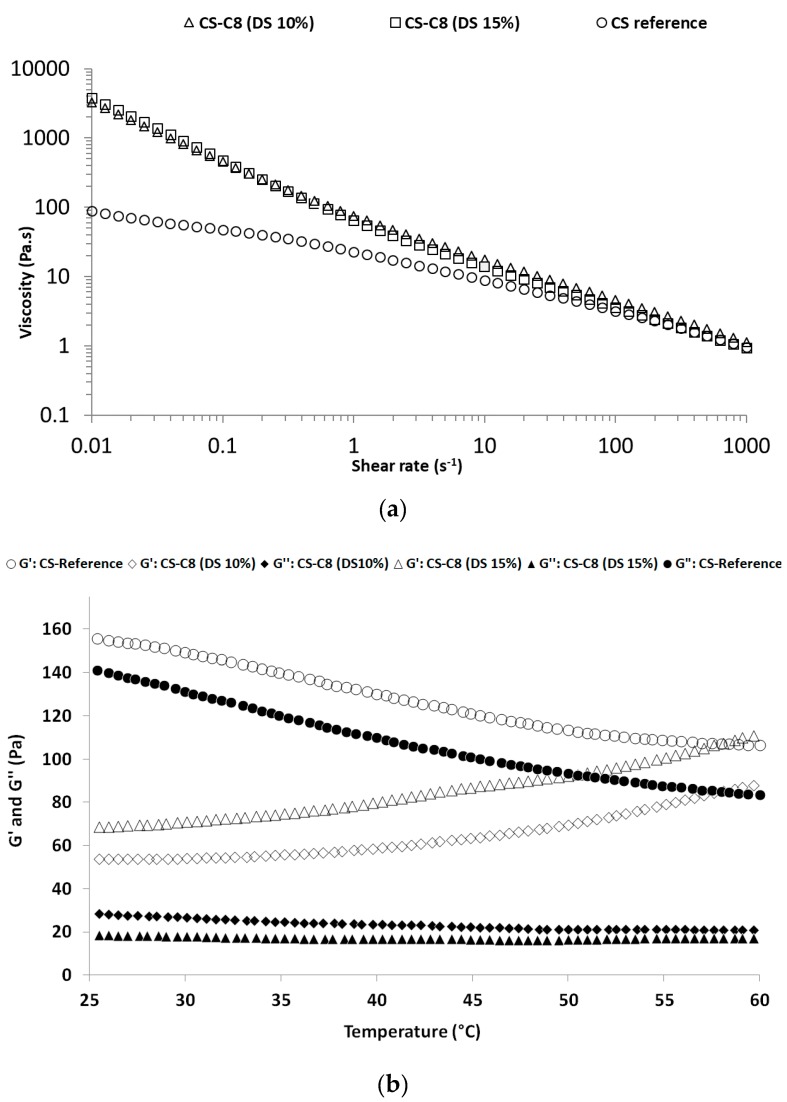
(**a**) Influence of the shear rate on the rheological curves of the solutions of native chitosan (CS-Reference) and alkyl-chitosans (DS of 10 and 15%) at 4% (*w/v*) in 1% acetic acid; (**b**) Plot of elastic modulus (G′) and loss modulus (G″) as a function of temperature on chitosan (CS-Reference) and alkyl-chitosans (DS of 10 and 15%) at 4% (*w/v*) in 1% acetic acid.

Rheological analyses were performed on native (CS-Reference) and modified chitosans (CS-C8 with DS of 10% and 15%) at different concentrations (2, 4, and 6%). Figure 2a presents the variation of viscosity as a function of shear rate for three samples considered as representative for their adhesive properties, namely the solutions at 4% (*w/v*) of native chitosan and alkyl-chitosans.

The rheograms show that all samples are non-Newtonian fluids, since the viscosity decreases with increasing shear rate, and the viscosity is higher for the solutions of alkyl-chitosans. The same tendency was reported in the literature [9,12]. Moreover, Desbrières [9] mentioned also that the increase in viscosity of alkyl-chitosans with the degree of substitution due to intermolecular hydrophobic interactions plays a major role in the gelation process and, thereby, confers a change in the physico-chemical (rheological) properties of the modified chitosans. The increase in viscosity with concentration suggests, in terms of adhesive application, that for alkyl-chitosans it is preferable to use concentrations lower than 6% (*w/v*) due to the difficulty in rolling out the adhesive on the adherends whatever the type of surface (wood or aluminium).

From Figure 2b, the viscoelastic modulus (G′) is larger than the viscous (or loss) modulus (G″), demonstrating a gel-like behavior. Moreover, for native chitosan, G′ and G″ decrease with increasing temperature, while for the alkylated chitosan (10 and 15%), G′ increases with temperature and G″ remains constant. The same tendency was already reported by Desbrières [9].

Due to concentration (4%, *w/v*) being far higher than the overlap concentration mentioned by Desbrières [9], which was lower than 2% (*w/v*) in the case of high-molecular-weight chitosan with a DD of 80%, the application of classical rheological models for the interpretation of flow behavior is not relevant. However, to obtain more information specially on the coating of adhesive fluids based on CS-C8 with concentrations up to 2% (*w/v*), the Ostwald-de-Waele empirical relationship was applied and the parameters of this model (flow index “n” and consistency index “k”) were determined. Therefore, the data shown in Table 3 provide an idea of the rheological behavior that could be linked to the processing behavior of both chitosan and alkyl-chitosans.

## 3. Discussion

During the last decades, chitosan has gained significant attention as a potent natural adhesive. At lower concentrations (<10% (*w/v*)), it offers competitive strength compared with synthetic adhesives such as phenol-formaldehyde. In this study, the application of alkyl-chitosan as a water-resistant adhesive was approached for the first time. Alkyl-chitosans were successfully synthesized by a Schiff reaction with an octanal (C8). The reaction was performed in mild conditions with no modification of the degree of acetylation. Two degrees of substitution, expressed as the molar ratio of substituted amine groups, were obtained during this study (10 and 15%). Moreover, the physicochemical properties of alkyl-chitosan-based adhesives were compared to those of native chitosan. The concentrations of both formulated chitosan and alkyl-chitosan ranged from 2 to 6% (*w/v*), which represents the best concentration area for high-molecular-weight chitosan [2,6,7].

The bonding properties obtained in this study with native chitosan in dry conditions were similar to those obtained by Reference [2,6] on wood and chemically treated aluminium double-lap specimens. In this study, the bond strength was better in native chitosan and on wood specimens compared with alkyl-chitosans and treated aluminium specimens, where a decrease of the bond strength was observed, probably due to the increase of hydrophobic interaction and high viscosity obtained after the alkylation process.

Nevertheless, the series bonded with alkyl-chitosan with a DS of 15% exhibited improved water resistance conserving its initial shear strength at 0.7 MPa. We can conclude that alkylation increases water resistance compared with the unmodified adhesive based on chitosan, especially when the degree of substitution is higher than 10%. We assume that a loss of adhesive properties can occur when the DS is higher than 15% and/or the alkyl chain is bigger than C8. In fact, the balance between adhesion/water resistance is correlated with the hydrophilic/lipophilic balance.

It is important to note that regarding the European standard EN 204, in particular the classification D4 (model n°2) of water-resistant wood adhesive, the bond strength of the adhesive should be equal to or greater than 8 MPa when samples are left 7 days at room temperature (23 ± 2 °C, 50.5% of relative humidity), then 3 h in a water bath at 20 ± 5 °C, followed by 7 days at room temperature. This value was not achieved during this study. However, this work demonstrates the potential for developing alkyl-chitosan derivatives as water-resistant adhesives in particular for wood adhesive applications. It will be interesting to test the bonding properties and water resistance of alkyl-chitosans at different concentrations and grafted with other aldehydes (C12, C20, C40...) with various degrees of substitution to reach the required bond strength.

## 4. Materials and Methods

### 4.1. Chemicals

Chemicals for the synthesis of alkyl-chitosan derivatives were as follows: high-molecular-weight chitosan (*M* = 300,000 g·mol^−1^), 82% deacetylated and derived from chitin of shrimp shells (Sigma Aldrich Co., St Louis, MO, USA); aldehyde (octanal, CH_3_(CH_2_)_6_CHO from Sigma Aldrich Co.); sodium hydroxide (NaOH, pellets from Sigma Aldrich Co.); sodium cyanoborohydride reagent grade 95% (NaBH_3_CN, Sigma Aldrich Co.); glacial acetic acid 99.5% (Sigma Aldrich Co.) and ethanol 96% (Sigma Aldrich Co.).

### 4.2. Synthesis of Alkyl-Chitosan

**Figure 3:**
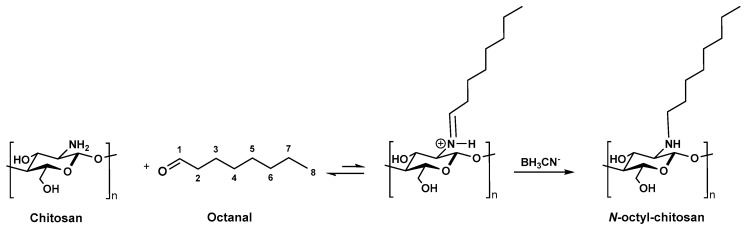
Synthesis of *N*-octyl-chitosan by alkylation process.

The alkyl-chitosan derivatives were obtained by reductive amination following the procedure previously described by References [8,12]. This is a specific method for creating a covalent bond between a substrate, namely an aldehyde (C8), and the amine function of the glucosamine unit of chitosan (Figure 3).

The alkylation reaction was processed as follows: 6 g of chitosan were dissolved in 450 mL of 0.2 M acetic acid (AcOH). When dissolution occurred, 180 mL of ethanol (EtOH) was added to allow the aldehyde used for the alkylation to be in a solvating medium. The pH was adjusted after complete dissolution to 5.1 to avoid the precipitation of the macromolecules, the optimal reaction pH ranges being between 4 and 8. The solution of the aldehyde in EtOH was added at the adequate ratio prior to an excess of sodium cyanoborohydride NaBH_3_CN (3 moles per chitosan monomol). The mixture was stirred during 24 h at room temperature, and the alkyl-chitosan was precipitated with EtOH. The pH was adjusted to 7 with a sodium hydroxide solution and the precipitate was washed with EtOH/water mixtures with increasing EtOH content from 70% (*v/v*) to 100%. The primary amino groups of chitosan undergo a Schiff reaction with aldehydes to yield the corresponding aldimines, which are converted to an alkyl derivative by reduction with NaBH_3_CN.

### 4.3. Structural Characterization of Chitosan and Alkyl-Chitosan

The ^1^H-NMR spectroscopy was used to characterize the chitosan and alkyl-chitosan derivatives in order to establish the deacetylation degree (DD) and the degree of substitution (DS). The ^1^H-NMR spectra were obtained on a Bruker Avance DRX 400 spectrometer (Fallanden, Switzerland) in D_2_O-HCl (pH~4), at a frequency of resonance of 400 MHz and temperature of 80 °C. The deacetylation degree (DD) and the degree of substitution (DS) were calculated from the ^1^H-NMR spectra by applying a previously described method [12].

### 4.4. Formulation of Chitosan- and Alkyl-Chitosans-Based Adhesive

The native polysaccharide and the modified ones were solubilized at concentrations ranging from 2 to 6% (*w/v*) in acetic acid 1% to 2% (*v/v*) (Sigma-Aldrich, 98.9%) at room temperature (20 °C) for 24 h under continuous stirring. The formulations from native chitosan were yellowish compared to the formulations of alkyl-chitosan that were transparent at 2% (*m/v*) or white viscous gel at 4 and 6% (*w/v*).

### 4.5. Adherend Preparation

The adherends used to prepare the wood specimens were softwood species of the European pine (*Pinus pinaster*), also known as maritime pine, purchased in a local market (Clermont-Ferrand, France). This wood species has often been used for adhesive characterization purposes. It is also widespread in many countries for wood applications. No specific treatment is used on *Pinus pinaster* softwood specimens before manufacturing.

Based on the results of Patel et al. [2], aluminium adherends were pre-treated according to the following protocol: a 1 mol/L NaOH treatment (1 h) followed by washing with mild detergent and storage overnight in 2% (*v/v*) acetic acid.

**Figure 4:**
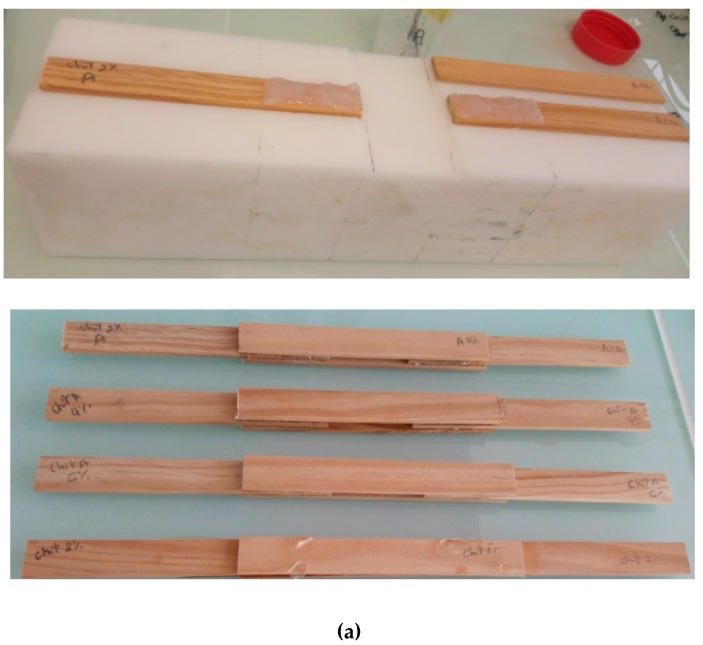

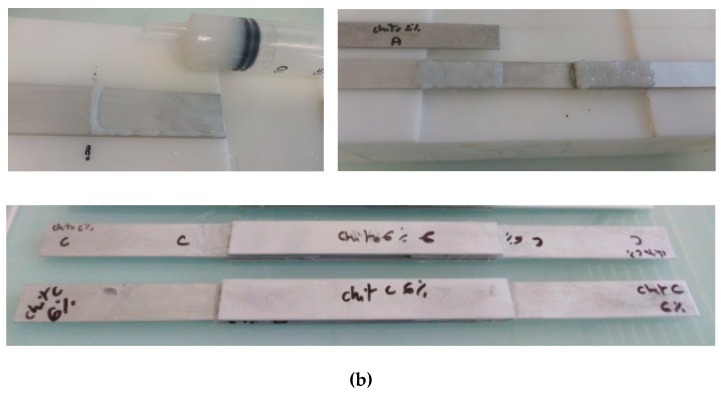
Illustration of bonded double-lap wood (**a**) and aluminium (**b**) specimens prepared for the mechanical characterization.

To evaluate the bonding properties of the alkyl-chitosan adhesive, double-lap treated wood (Figure 4a) and aluminium specimens (Figure 4b) were used following the method described by Patel et al. [2,6]. Each specimen is composed of four rectangular and identical plates (laps) bonded together following a previously described protocol [2,6]. The dimensions of the plates were 124 × 18 × 4 mm^3^, and the lap area was 50 × 18 = 900 mm² (Figure 4a,b). For each specimen, about 2.5 g of the formulated adhesive at different concentrations (2, 4, and 6%) and different DS (0, 10, and 15%) were poured using a syringe between two adherend laps. At first, the areas 1/2 and 1/3 were assembled, bonded, and dried during 6 h at 50 °C without applying any pressure. Then areas 2/4 and 3/4 were bonded, as well, to form the double lap systems which were dried during 24 h at 50 °C and were kept at 23 °C and 50% relative humidity before mechanical analyses.

### 4.6. Rheological Measurements

Apparent viscosity measurements were carried out as it was described by Reference [16] using double concentric cylinder geometry and parallel plate geometry with a stress-controlled rheometer AR-G2 (TA Instruments, Guyancourt, France) equipped with a Peltier temperature control system. The temperature was set at 20 °C and the viscosity was monitored in the range of shear rate from 10^−2^ to 10^3^ (s^−1^). Each solution of chitosan was measured twice. Dynamic viscoelastic measurements were also performed using the same rheometer on formulated chitosans, using a parallel plate shear mode to measure the storage modulus (or elastic modulus), G′ (Pa), and the loss modulus (or viscous modulus), G″ (Pa). The linear viscoelastic region was determined using strain sweep tests. Temperature ramp tests were carried out between 25 °C and 60 °C at 5% strain and 5 Hz to analyze the effect of temperature. A total of 3 mL of hexadecan were poured onto the samples before each analysis to avoid water and acetic acid evaporations. Viscosity data were collected and analyzed using the Rheology Advance software package. The curve modelling was achieved using the power law Ostwald-de-Waele relationship (1):

*τ* = k · *γ*^n^ (1)

where *τ* is the shear stress (Pa), obtained by multiplication of the apparent viscosity and the shear rate; *γ* is the shear rate (s^−1^), “k”, the consistency index (Pa·s^n^), and “n” the flow behavior index (for pseudo plastic or shear-thinning fluid n < 1).

### 4.7. Mechanical Characterization

The mechanical characterization of the different bonded specimens was performed following the method described by Patel et al. [2] using a Zwick Roell testing machine (Zwick GmbH & Co., Ulm, Germany) with a crosshead speed of 0.05 mm/s. The testxpert V11.02 (Zwick GmbH & Co., Ulm, Germany) was used for recording the force versus displacement of the moving grip. The bond strength of the adhesive (σ) (MPa) was deduced by calculating the shear stress using the following Equation (2):

σ = F/2A(2)

where F is the applied force (N) and A is the lap area (mm²). Each experiment was repeated three times, and the average and corresponding standard deviation were calculated in each case.

Between each adhesive formulation, it was not possible to obtain the same thickness of bonded joints. Patel et al. [2], working on aluminium adherends, proposed comparing the value of the maximum shear stress obtained with the Volkersen’s model using the following Equations (3)–(5):

τ(0) = (G2σ0)/sinh〖(λL_2_)e_2_λE_1_〗(1 + cosh(λL_2_)) (3)

with

λ = √((2G_2_)/(e_1_e_2_E_1_)) (4)

and

σ_0_ = F/S (5)

where F is the applied force (N), S is the cross section of the adherend (m²), σ_0_ the axial stress, e_1_(m) the thickness of the adherend, e_2_(m) the adhesive thickness, L_2_ is 0.05 m corresponding to the lap length, and E_1_ is the Young modulus of the adherends (72 GPa for aluminium and 12 GPa for wood). The shear modulus G_2_ of the adhesive was determined by Patel et al. [2] at 0.76 GPa.

### 4.8. Water Resistance Essay

The water-resistance analysis was performed on wood bonded samples with native chitosan and alkylchitosan with DS of 10 and 15%. The used model was inspired on the European current standard EN 204-D4 (model n°2) and has been employed in the literature [2,5]. The test was realized in triplicate, consisted in leaving the bonded samples 7 days at room temperature at 23 ± 2 °C, 50.5% of relative humidity (RH), then 3 h in a water bath at 20 ± 5 °C, followed by 7 days at room temperature (20 ± 2 °C) and 50 ± 5% of RH. Mechanical characterization was performed after this test on the cited specimens in triplicate.

## Figures and Tables

**Table 1:** Bond strength and shear stress of native and alkyl-chitosan adhesives on treated aluminium and wood adherends; e_Al_ and e_W_ are the adhesive thicknesses obtained on aluminium and wood adherends, respectively.

Concentration of Formulated Adhesive % (*w/v*)	Bond Strength		Shear Stress			
	Aluminium	Wood	Aluminium		Wood	
	σ (MPa)	σ (MPa)	e_Al_ (mm)	τ (0) (MPa)	e_W_ (mm)	τ (0) (MPa)
CS2	0.22 ± 0.07	1.25 ± 0.01	0.10 ± 0.02	31.85 ± 4.33	0.5 ± 0.01	38.84 ± 1.65
CS4	0.23 ± 0.07	2.55 ± 0.02	0.07 ± 0.02	61.87 ± 11.40	0.79 ± 0.01	39.53 ± 1.15
CS6	0.38 ± 0.49	1.92 ± 0.02	0.30 ± 0.20	26.24 ± 19.00	0.80± 0.05	30.09 ± 2.81
CS-C8 (DS * 10%) 2	0.37 ± 0.02	1.05 ± 0.01	0.53 ± 0.01	4.96 ± 0.10	0.70 ± 0.01	20.25 ± 0.44
CS-C8 (DS * 10%) 4	0.25 ± 0.03	1.30 ± 0.09	0.80 ± 0.05	1.82 ± 0.09	1.00 ± 0.05	13.45 ± 0.96
CS-C8 (DS * 10%) 6	0.23 ± 0.06	0.80 ± 0.09	1.50 ± 0.10	0.7 ± 0.24	1.24 ± 0.02	6.43 ± 0.16
CS-C8 (DS * 15%) 2	0.48 ± 0.07	0.62 ± 0.97	0.55 ± 0.15	6.65 ± 1.89	0.7 ± 0.01	11.71 ± 0.11
CS-C8 (DS * 15%) 4	0.23 ± 0.07	0.72 ± 0.53	0.72 ± 0.07	4.07 ± 1.24	1.00 ± 0.05	8.05 ± 0.52
CS-C8 (DS * 15 %) 6	0.23 ± 0.07	0.93 ± 0.24	1.00 ± 0.25	2.74 ± 1.84	1.15 ± 0.05	6.52 ± 0.39

* DS: Degree of substitution.

**Table 2:** Mechanical properties of native and alkyl-chitosan at 4% (*w/v*) before and after immersion test of wood bonded specimens. σ corresponds to the bond strength (MPa)

	Before	After
Formulated Adhesive at 4% (*w/v*)	σ (MPa)	σ (MPa)
CS-Reference	2.55 ± 0.02	0.28 ± 0.02
CS-C8 (DS 10%)	1.30 ± 0.09	0.22 ± 0.01
CS-C8 (DS 15%)	0.72 ± 0.53	0.73 ± 0.18

**Table 3:** Rheological characteristics of chitosan and alkyl-chitosans at 4% (*w/v*) using the Ostwald-de-Waele relationship. (“n” represents flow index and “k” represents the consistency index).

Adhesive at 4% (*w/v*)	n	k	R^2^
CS-Reference	0.62 ± 0.01	18.61 ± 0.76	0.99
CS-C8 10%	0.29 ± 0.02	106.56 ± 6.94	0.96
CS-C8 15%	0.25 ± 0.02	97.99 ± 7.10	0.93

## References

[1] Mamiński M.L., Borysiuk P., Zado A. (2008). Study on the water resistance of plywood bonded with UF-glutaraldehyde adhesive. Holz Roh. Werkst..

[2] Patel A.K., Michaud P., Petit E., de Baynast H., Grédiac M., Mathias J.-D. (2012). Development of a chitosan-based adhesive. Application to wood bonding. J. Appl. Polym. Sci..

[3] Mati-Baouche N., Elchinger P.-H., de Baynast H., Pierre G., Delattre C., Michaud P. (2014). Chitosan as an adhesive. Eur. Polym. J..

[4] Yamada K., Chen T., Kumar G., Vesnovsky O., Topoleski L.D.T., Payne G.F. (2000). Chitosan based water-resistant adhesive. Analogy to mussel glue. Biomacromolecules.

[5] Umemura K., Inoue A., Kawai S. (2003). Development of new natural polymer-based wood adhesives I: Dry bond strength and water resistance of konjac glucomannan, chitosan, and their composites. J. Wood Sci..

[6] Patel A.K., Michaud P., de Baynast H., Grédiac M., Mathias J.-D. (2012). Preparation of chitosan-based adhesives and assessment of their mechanical properties. J. Appl. Polym. Sci..

[7] Mati-Baouche N., de Baynast H., Lebert A., Sun S., Sacristan Lopez-Mingo C.J., Leclaire P., Michaud P. (2014). Mechanical, thermal and acoustical characterizations of an insulating bio-based composite made from sunflower stalks particles and chitosan. Indus. Crops Prod..

[8] Yalpani M., Hall L.D. (1984). Some chemical and analytical aspects of polysaccharide. Modifications. Formation of branched-chain, soluble chitosan derivatives. Macromol.

[9] Desbrieres J. (2004). Autoassociative natural polymer derivatives: The alkylchitosans. Rheological behaviour and temperature stability. Polymer.

[10] Laroche C., Delattre C., Mati-Baouche N., Salah R., Ursu A.V., Moulti-Mati F., Michaud P., Guillaume P. (2017). Bioactivity of chitosan and its derivatives. Curr. Org. Chem..

[11] Lepoittevin B., Elzein T., Dragoe D., Bejjani A., Lemée F., Levillain J., Bazin P., Roger P., Dez I. (2019). Hydrophobization of chitosan films by surface grafting with fluorinated polymer brushes. Carborhydr. Polym..

[12] Desbrieres J., Martinez C., Rinaudo M. (1996). Hydrophobic derivatives of chitosan: Characterization and rheological behaviour. Int. J. Biol. Macromol..

[13] Aranaz I., Harris R., Heras A. (2010). Chitosan amphiphilic derivatives. Chemistry and applications. Curr. Org. Chem..

[14] Nicu R., Lupei M., Balan T., Bobu E. (2013). Alkyl-chitosan as paper coating material to improve water barrier properties. Cellul. Chem. Technol..

[15] Bobu E., Nicu R., Lupei M., Ciolacu F., Desbrieres J. (2011). Synthesis and characterization of N-alkyl chitosan for papermaking application. Cellul. Chem. Technol..

[16] Mati-Baouche N., de Baynast H., Vial C., Audonnet F., Sun S., Petit E., Pennec F., Prevot V., Michaud P. (2015). Physico-chemical, thermal, and mechanical approaches for the characterization of solubilized and solid state chitosans. J. Appl. Polym. Sci..

